# The limits of sustainability of peat as a soilless media for agriculture

**DOI:** 10.3389/fpls.2026.1856037

**Published:** 2026-07-10

**Authors:** James Altland, Bruce Bugbee

**Affiliations:** 1 Application Technology Research Unit, Agricultural Research Service, US Department of Agriculture, Wooster, OH, United States; 2 Crop Physiology Laboratory, Utah State University, Logan, UT, United States

**Keywords:** carbon footprint, global warming potential, growing media, horticulture, peatland, restoration

## Abstract

Soilless media has long been used to improve fertilizer and water use efficiency of high-value crops in containers, and global demand for these media is predicted to increase 250% to 400% in the next 25 years. The environmental impact of all soilless media products warrants detailed analysis. The unique properties of peat make it the most widely used product. Peatlands are vital carbon stores and the historical degradation of European bogs has prompted regulations restricting peat extraction. Unlike Europe, North American peatlands—particularly in Canada—are largely pristine and have the potential to be sustainably managed. A focus on restoration, coupled with limited human interaction in remote peatlands, means that Canadian peatlands are currently accumulating carbon faster than the rate of extraction for containerized crop production. However, the steadily increasing use of soilless media for high-input agriculture may drive-up international demand for North American peat, requiring careful management to maintain sustainability. Several alternative products have been studied but our review of seven life cycle assessments reveals that their environmental impact is similar to or greater than peat. The expansion of high-input agriculture means that multiple soilless media components will be needed to feed our growing population.

## Introduction

Peatlands cover only 3% of the Earth’s land area but contain more carbon than all the vegetation on the planet (Xu et al., 2018). A millennium of draining peatlands for settlements and agriculture (Borger, 1992) and three-hundred years of over-extraction of peat for energy in Europe has led Germany, Ireland, the United Kingdom, Norway, Finland, and Switzerland to phase-out peat extraction (Hirschler and Osterburg, 2022; Brioche, 2023; Carlile, 1999).

Unlike Europe, North America has minimally burned peat for energy and there are vast undisturbed peatlands in Canada. Extraction of Canadian peat to supply horticulture accounts for only 1% of all drained or degraded peatlands^1^ and just 0.03% of the total peatland area. Using modern approaches, these peatlands can quickly be restored to carbon-sequestering and peat-accumulating ecosystems (Grace, 2004).

But there has been a wide-spread perception of unsustainability. The New York Times reported that “*Peat extraction releases substantial carbon dioxide contributing to climate change*” and that “*the consequences of its continued use … is too high*” (Roach, 2022). National Geographic claimed that horticultural substrates are “*dirt-less sterile blends of exotic mosses, fibers, and minerals, ingredients that hide lung disease, water waste, and a whopping carbon footprint*.” (Etherton, 2022). Popular gardening magazines and blogs repeat the message to consumers with vague accusations about the lack of peat sustainability (Nay, 2023; Nosowitz and Tychonievich, 2024). Schmilewski (2014) pointed out that “*debates are often based on biased approaches by stakeholder groups*”.

Here we:

1. review global carbon storage and emissions associated with horticultural peat extracted from boreal or temperate peatlands
2. review the global distribution and health of peatlands
3. quantify peat growth rate and peatland restoration
4. review the environmental impact of proposed alternatives, and
5. describe the global demand for soilless media and its implications for the future of peat harvesting.

## Global carbon storage and emissions of peatlands

**Figure 1:**
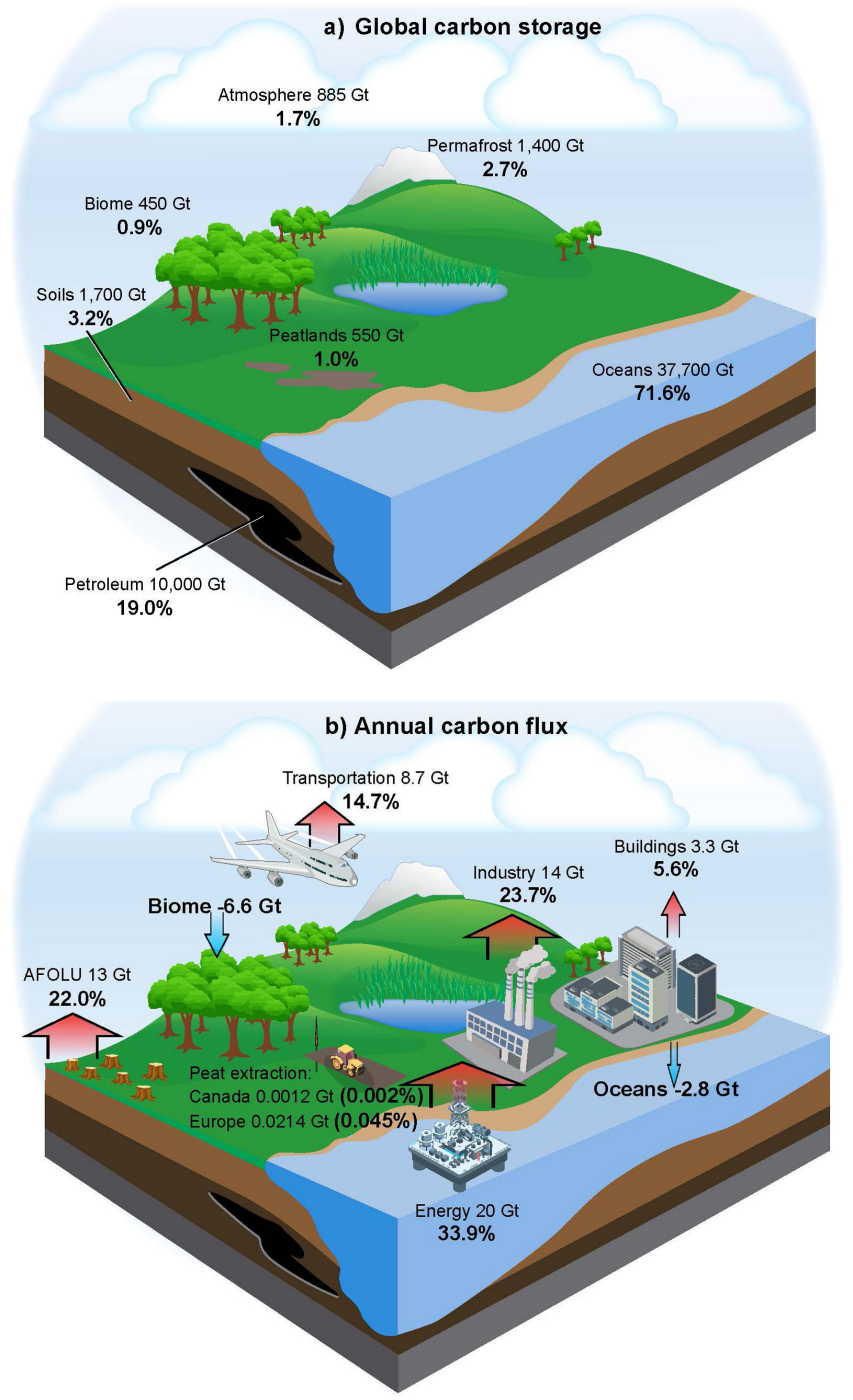
**(a)** Global storage of carbon in natural systems (Gt, gigatons) and **(b)** Annual flux of carbon from human activities and sequestration by natural processes (Gt CO_2_-eq per year) (Hirschler and Osterburg, 2022; Cleary et al, 2005; IPCC, 2023).

The primary concern about the extraction of peat for horticulture is the effect on atmospheric CO_2_. A summary of global carbon storage and flux puts peat extraction in perspective. At a CO_2_ concentration of 420 ppm in 2023 (NOAA, 2024) our atmosphere stores 885 Gt CO_2_-eq^2^,^3^ (**Figure 1a**). Peatlands contain 500 to 600 Gt CO_2_-eq globally (Gorham et al., 2012) and have been accumulating 0.04 to 0.07 Gt CO_2_-eq per year (Roulet, 2000). The 1.19 × 10^6^ km^2^ of Canadian peatlands^4^ have been estimated to contain between 154 and 163 Gt CO_2_-eq (Gorham et al, 2012; Roulet, 2000) with a current accumulation rate estimated at 0.03 Gt CO_2_-eq per year (Roulet et al., 2007). The sum of global carbon emissions from human activities is 59 Gt CO_2_-eq per year (IPCC, 2023) (**Figure 1b**) resulting from energy production, industry, transportation, buildings, and other uses. Of this total, agriculture, forestry, and other land uses (AFOLU, a widely used acronym) releases 13 Gt CO_2_-eq per year. Canadian peat harvests for horticulture have occurred on 360 km^2^, with an active footprint of just 250 km^2^, and account for 0.0012 Gt CO_2_-eq per year (Cleary et al., 2005), just 0.002% of the global carbon flux.

## Global distribution and health of peatlands

While accumulation of C in peatlands is slow, their storage capacity is substantial and reversal from a sink to a source could dramatically increase atmospheric CO_2_.

**Figure 2:**
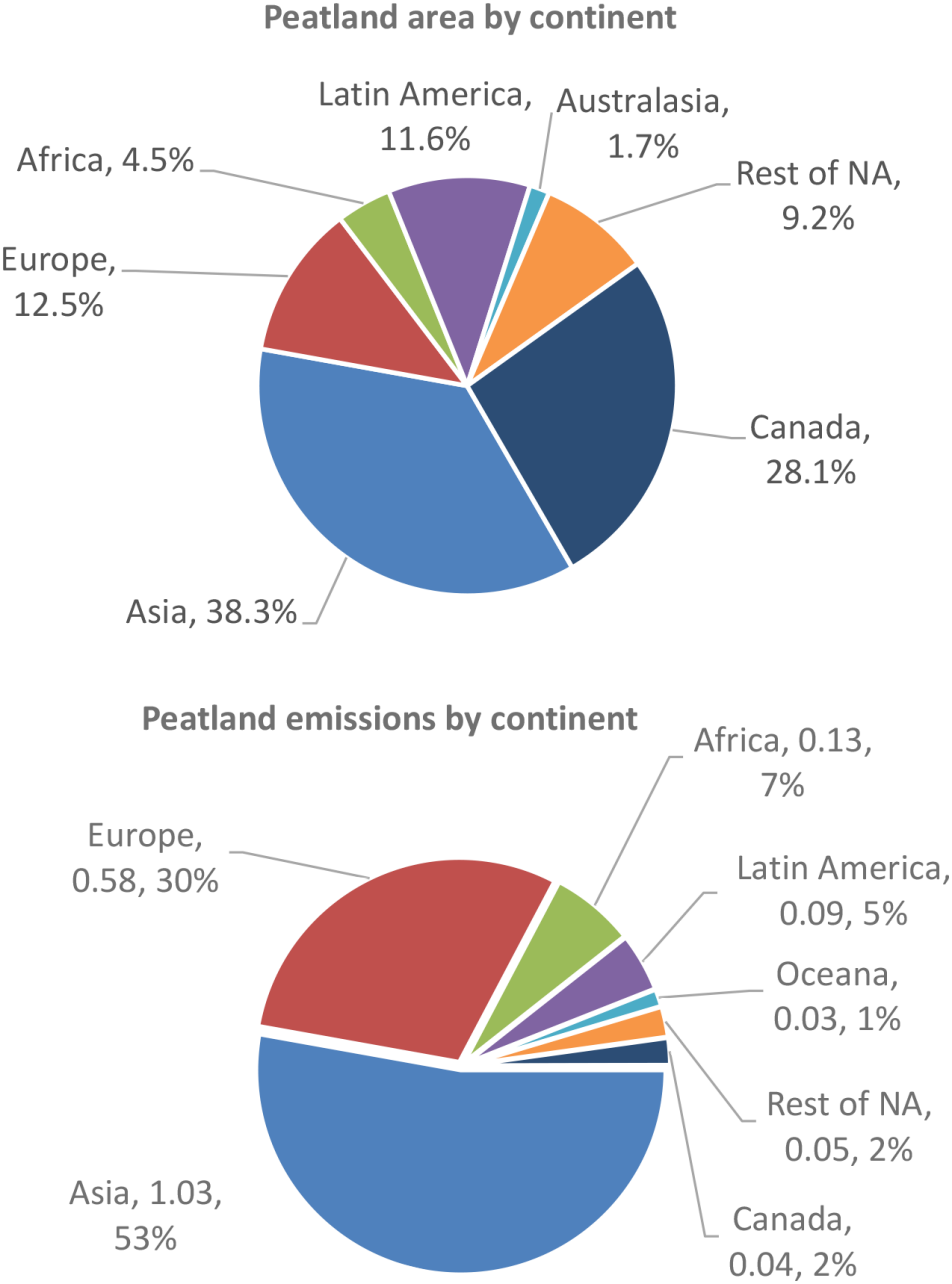
Global distribution of peatlands in land area (Xu et al, 2018) and carbon emissions (Gt CO_2_-eq per year, and % of total) from degraded peatlands (Kopansky et al., 2022) by continent.

Of the world’s 4.23 × 10^6^ km^2^ peatlands (Xu et al., 2014), approximately 12% are considered degraded^5^ (Kopansky et al., 2022). The 0.6 × 10^6^ km^2^ of European peatlands are the most degraded in the world (**Figure 2**). Estimates indicate nearly 50% are degraded and 10% are completely lost (Joosten and Clarke, 2002; Kopansky et al., 2022). Degradation decreases with latitude: arctic zones are far less degraded (1%) than southern boreal and temperate zones (68%). Use of peatlands in Europe began as early as the 10^th^ century (Borger, 1992) for salt making, farming new land, and extraction for fuel. Borger (1992) characterized the history of peatlands in Europe as “a history of their destruction”. Ireland, Poland, Denmark, The Netherlands, and Germany have all drained greater than 80% of their peatlands (Kopansky et al., 2022). These permanently drained peatlands now emit about 0.6 Gt CO_2_-eq per year (Kopansky et al., 2022) and account for approximately 25% of global agricultural CO_2_-eq emissions despite only making up approximately 3% of global agricultural land area (Kopansky et al., 2022).

North America has 1.4 × 10^6^ km^2^ of peatlands (1.19 in Canada and 0.19 the US). The vastness of Canadian peatlands is hard to overstate. As a frame of reference, the 1.19 × 10^6^ km^2^ of Canadian peatlands are approximately double the land area of Texas, while the 360 km^2^ of peatlands extracted for horticulture would fit within the Austin city limits. The boreal and temperate forest regions are the most important to agriculture. Approximately 1% of Canadian peatlands, or 0.02 × 10^6^ km^2^, are considered degraded (Kopansky et al., 2022), primarily due to permanent drainage for row crops and pastures (63%) followed by infrastructure for oil and gas mining (17%) (Kopansky et al., 2022). These degraded peatlands emit approximately 0.09 Gt CO_2_-eq per year (Kopansky et al., 2022).

This permanent drainage is far different from temporary drainage for extracting horticultural peat. Peatlands that have been temporarily drained can be restored, a routine practice by Canadian horticultural peat extractors for more than 20 years (González and Rochefort, 2014).

## Peat growth rate and peatland restoration

Sphagnum mosses in northern peatlands are estimated to accumulate from 0.3 to 0.7 mm peat per year (Vitt, 1994). If a depth of 1 m were extracted, it would take 1400 to 3300 years to replace the peat in that location. This analysis leads to the concern that peatlands cannot be restored in our lifetime, but Canadian peat bogs are collectively growing at a greater rate than they are being extracted. The ratio of accumulation to extraction can be derived in several ways – each of which indicates that accumulation is greater than extraction.

1. Atmospheric CO_2_-eq release from peat extracted for horticulture in 2000 was calculated to be 0.0012 Gt CO_2_-eq (Cleary et al., 2005). This calculation accounts for CO_2_-eq release from “cradle to grave”, including changes in land use, extraction, transportation, and complete oxidation of the peat. Net C accumulation in Canadian peatlands was estimated at 0.03 Gt CO_2_-eq per year (Roulet, 2000). By this metric, accumulation was 25 times more rapid than extraction.
2. Assuming a mass of 0.4 kg m^-2^ y^-1^ dry peat accumulation (548 Mt y^-1^ across 1.37 × 10^6^ km^2^ of peatlands) (Zoltai, 1991), and an annual harvest of 1.2 Mt dry peat for horticulture, Canadian peat is accumulating 457 times faster than extractions (548 Mt accumulation ÷ 1.2 Mt extraction).
3. If northern peatlands accumulate a 0.3 to 0.7 mm peat per year (Vitt, 1994), across the 1.37 × 10^6^ km^2^ of Canadian peatlands a volume of 411 to 959 Mm^3^ is produced. This is 34 to 80 times greater than the 12 Mm^3^ peat that was extracted for horticulture in 2023 (Hullett, 2023).
4. Canadian peat is typically harvested to a depth of 7 cm per yr, so that each cubic meter of peat originates from approximately 14.3 m^2^ peatland. A restored peatland can sequester 78 g m^-2^ y^-1^ C^27^, or 1.12 kg C per year on the extracted 14.3 m^2^. Assuming extracted horticultural peat eventually stabilizes at a decomposition rate of 1% per year (Conant et al., 2011), a cubic meter would release 1.83 kg per year CO_2_ (0.5 kg per yr C). The 1.12 kg per yr C sequestered is more than twice the 0.5 kg per yr C lost to decomposition from the harvested peat.

Global demand for soilless media is predicted to increase 250% to 400% by the year 2050 (Blok et al., 2021; Nguyen et al., 2026). Nguyen et al. (2026) project a 10% increase in Canadian peat harvests by 2050 with projections of other raw materials being used to meet demand. Even assuming peat harvests from Canada were to increase by the same percentage as global demand (4x of 2022), peat accumulation would still outpace peat harvests as the calculated peat accumulations rates range from 25x to 457x.

## Impact of extraction on peatland ecosystems

Peat extraction for horticulture requires drainage of the peatland and complete removal of surface vegetation. Within the boundaries of the extraction site, there is complete loss of above-ground biodiversity. More important may be the impacts of extraction on hydrological services that can impact regional watersheds beyond the extraction site. Watersheds downstream of peat extraction experience elevated dissolved inorganic nitrogen and iron loads, discharge dependent shifts in dissolved organic matter, and elevated eutrophication risk during high flow periods (Sääksjärvi, et al. (2016); Frei et al., 2024). Ditch networks used to drain peatlands for harvest increase downstream suspended solids and transiently elevate aluminum, causing chronic sediment stress (Nieminen et al., 2018). Peat harvesting shrinks on site water (precipitation) storage that exacerbates hydrograph flashiness, or the frequency and rapidity with which a stream or river’s water level and flow spike after a rain event (Haapalehto, et al., 2014). Collectively, peat harvesting and its drainage infrastructure reduce local water availability (via lowered water tables), degrade water quality (nutrients, metals, humic substances, suspended solids), and compromise hydrological services (storm moderation, carbon retention) at catchment scales.

## Post harvest restoration on peatland ecosystems

Across temperate Sphagnum peatlands, active restoration processes like the moss layer transfer technique (MLTT) accelerate vegetation recovery. Sphagnum cover approaches pre disturbance levels within 30 to 35 years, with many peatland specialists recovering in 20 to 25 years (Allan et al., 2024). Long term assessments of active restoration processes like the MLTT demonstrate sustained recolonization of Sphagnum and improvements in plant assemblages at post extraction sites, supporting MLTT as a robust approach for restoring biodiversity (Breton et al., 2026). Other research shows more rapid recovery with active rewetting and reestablishment of Sphagnum communities resulting in plant and invertebrate richness approaching reference sites within ~10 years (Everson et al. 2026). Elo et al. (2015) showed that blocking drains raises Odonata abundance and richness within three years while Stückler et al. (2024) showed that created peat pools in rewetted sites provide breeding habitat for amphibians and reptiles, both of which were documented immediately in the first season after restoration. Overall, rising water tables and Sphagnum dominance favors specialist assemblages, strengthening resilience of recovered biodiversity to climate variability (Swenson et al., 2019; Beadle et al., 2015).

Restoration that blocks drains and reestablishes saturation measurably increases water storage and attenuates stormflow, reducing peak discharge and runoff coefficients within 0 to 4 years (Gatis et al., 2023; Karimi et al., 2025). Water tables rise and stabilize, moving pore water chemistry toward pristine conditions; however, short term nutrient leaching (N, P) can increase for more than 5 years post restoration, implying a lag before water quality benefits fully manifest (Haapalehto, et al., 2014). Restoration effects on carbon related water quality are mixed. DOC loads may not decline immediately, and methane fluxes can increase from newly rewetted pools, even as stormflow moderation improves (Gatis et al., 2023). At larger spatial scales, process-based analyses show canal blocking produces modest mean rises in water table (~1.5 cm on annual averages) with efficacy limited to about 600 m from canals and strongly dependent on peat hydraulic conductivity (Urzainki et al., 2023). Overall, evidence supports restoration as effective for rebuilding water availability (higher, more stable water tables) and hydrological services (flood attenuation), with water quality gains emerging over longer time horizons.

Prompt restoration of harvested bogs can return them to a net carbon sink (Nugent et al., 2018) if waterlogged conditions and plant diversity are reestablished (Loisel and Gallego-Sala, 2022). Rochefort (2000) defines the goals of northern peatland (specifically in Canada) restoration as establishing sphagnum or brown mosses as the dominate plant cover, reestablishing the diplotelmic hydrological layers^6^, and a return of long-term functionality that ensures peat accumulation and resistance to biological invasion.

The cumulative peat growth rate across Canada, coupled with modern approaches to peatland restoration, indicates that peat use for North American horticulture is sustainable at current extraction rates.

***Sidebar: Modern approaches to peatland restoration**. The moss layer transfer technique (MLTT) was developed 30 years ago to improve restoration of harvested peat bogs. Although limited to a single site, Nugent et al. (2018) compared the net ecosystem exchange (NEE, i.e. CO_2_ flux), methane, and dissolved organic caron (DOC) in a post-restoration peatland (restored 14 years prior to the study) using the MLTT to an intact peatland within the same climate zone and a similar vegetation assemblage over three years. They found that the restored peatland had more negative NEE compared to the intact peatland (-90 vs. -73 g C m^−2^, where more negative numbers mean greater C sequestration) across the three-year study period. The restored peatlands also resulted in less emitted methane (4.4 vs. 6.0 g C m^−2^) and DOC (6.9 vs. 17 g C m^−2^) for a total net ecosystem carbon balance of -78 g C m^−2^ on the restored sites and -50 g C m^−2^ at the intact reference site. They concluded that C uptake can be re‐established within 14 years of restoration with the MLTT.*

*On a broader scale, González and Rochefort (2014) evaluated the success of 53 peatland restoration projects across eastern Canada that included 246 permanent plots that ranged from 3 to 15 years post restoration. Successful restoration, defined as dominance of a Sphagnum carpet, was found in 54% of all plots. Growth and spread of Sphagnum carpets can be slow, sometimes taking years to develop completely or to transition from a Polytrichum-dominated site to a Sphagnum-dominated site. González and Rochefort (2014) recommend waiting at least 10 years to decide if a restoration effort is successful, while acknowledging that many regulatory or permitting actions will require much shorter reporting periods. Likewise, Mander et al. (2024) used a conceptual framework drawing on literature from throughout Europe and North America to conclude that extracted peatlands restored to a functioning bog would become C neutral in 20 years and then would sequester 0.22 t h^-1^ C per year thereafter.*

## Europe and North America have dramatically different peatland health

North America has almost three-fold the area of peatlands compared to Europe, but they are 90% less degraded with 85% less CO_2_-eq emissions (**Table 1**).

**Table 1:** Comparison of peatland size, degradation, and CO_2_-eq emissions in Europe and North America (Kopansky et al., 2022).

	Europe	North America	North America to Europe ratio
Total land area (km2)	10,000,000	24,709,000	2.5
Total peatland area (km2)	587,556	1,382,000	2.4
Total degraded peatland area (km2)	272,626	28,476	0.1
% of peatland degraded	46.4	1.8	0.04
CO2-eq emissions from degraded peatlands (Gt CO2-eq per year)	0.58	0.08	0.14

The annual rate of peat extraction is five-fold greater in Europe. From 2013 to 2017, Europe extracted 29 Mm^3^ per year for horticulture; and surprisingly, also extracted 37 Mm^3^ per year for energy (Hirschler and Osterburg, 2022). While the use of peat for energy has been reduced by 57% from 2017 to 2023, Europe still burned 11.7 Mm^3^ ^7^ in 2023 (Eurostat, 2025). Canada extracted 12 Mm^3^ for horticulture in 2023 and none for energy (Hullett, 2023).

***Sidebar: Population and peatland degradation.** Degraded peatlands are those with a lowered water table (i.e. drained) that disrupts the peatland’s natural waterlogged anoxic state (Joosten and Clarke, 2002). The primary agent causing drainage and degradation of peatlands for the past 1000 years (Borger, 1992) has been human settlement and our need for farmland, dwellings, and infrastructure. The history of human settlement helps to explain the difference in peatland health between Canada and Europe. The population of Canada in 1800 was 1% of that in Europe (0.5 vs. 50 M people) (Statista, 2024). Even after 1900, rate of population growth in the ten European countries with the greatest peatland area (Estonia, Finland, Germany, Ireland, Latvia, the Netherlands, Norway, Poland, Sweden, and the United Kingdom) was greater than Canada so that by 2020 differences grew further (38 vs. 236 M people). And Canada has more than 4 times the land area of the ten European countries (9.98 × 10^6^ km^2^ vs. 2.32 × 10^6^ km^2^). The combined population of these ten European countries in the year 1100 was 9.9 M (data not shown), thus the population density for the European countries in the year 1100 (4.4 people per km^-2^) was greater than the population density of Canada today (3.7 people per km^-2^). Considering the relationship between peatland degradation and human population, it’s clear why there exists such disparity between peatland health in Canada versus Europe.*

## Comparison of alternatives based on life cycle assessment

Multiple products have been used as peat substitutes (Gruda, 2019): coconut husk (coir pith), mineral wool^8^, perlite, wood fiber, bark, sawdust, and compost. Barrett et al. (2016) provided a thorough review of the environmental costs and benefits of these emerging alternatives. All media have environmental impacts.

The environmental effects of these products are best determined by life cycle assessments (LCAs), which are quantitative tools for understanding the impact of products or services on the environment, human health, and society. All publicly available LCAs for growing media that provide assessment of peat and other materials as a single component were reviewed.

Cleary et al. (2005) conducted the most comprehensive LCA of Canadian peat and included harvests from 1990 to 2000 (Cleary et al., 2005). During this interval, the Canadian peat industry extracted 1.3 Mt peat. Cleary et al. (2005) estimated a total GWP of 0.893 Mt CO_2_-eq and attributed this total to land use (15%), extraction and processing (4%), transport to market (10%), and immediate, complete oxidation of the extracted peat (71%). Assuming a fresh harvested density of 0.14 t m^-3^, the average annual harvest described by Cleary et al. (2005) would have yielded approximately 8.9 Mm^3^ of peat. Assuming a 50% moisture content of the bailed peat, this would have resulted in a GWP of 50 kg CO_2_-eq per m^3^ of peat.

***Sidebar: The assumption of immediate oxidation to CO_2_ warrants further analysis**. The long-term stability of organic carbon in peat is an important component in the calculation of GWP. Even after harvest, peat degrades slowly and its carbon is conserved for many decades after use (Sharma et al., 2024).*

*Sharma et al. (2024) quantified degradation rates of 28 types of horticultural peat after pH adjustment and fertilization and reported a degradation rate of 0.15 ± 0.017 mg CO_2_-C g^-1^d^-1^. This rate is equivalent to 5.5% degradation per year. But this was based on the degradation rate after only 4 days. Degradation rates typically decrease over time, as the more easily degradable compounds are degraded before lignin and other complex organic molecules (Leifeld et al., 2012). In a 90-day study, Sharma et al. (2025a) reported degradation rates of peat of 6.6% ± 3.1% per year. But the authors noted that this rate likely included CO_2_ efflux from slow degradation of limestone amendments. Accounting for limestone contributions, they estimated a yearly degradation rate of 3.3% ± 1.6%. In a recent paper, Sharma et al. (2025b) reported the potential for a slightly higher peat heterotrophic degradation rate because the roots of petunia might accelerate the degradation of peat.*

*These estimates provide a more accurate foundation for comparing the environmental footprint of peat-based growing media to alternative media. By assuming immediate oxidation, the Cleary et al. (2005) study appears to have significantly overestimated the GWP of horticultural peat.*

*Peat can ultimately be recycled to landscapes to improve soil tilth. Similarly, the stability of organic matter in soils is crucial to understanding climate change impacts (Galluzzi et al., 2025; Lehmann and Kleber, 2015). Peat gradually breaks down to humus, which is a relatively stable form of soil organic matter. It can then be physically bound to soil particulate matter (Lehmann and Kleber, 2015). Because this process occurs over decadal to century time scales, it is difficult to predict long-term soil organic carbon decay from short-term laboratory studies (Conant et al., 2011; Giardina and Ryan, 2000). Nonetheless, estimates (Raviv et al., 2019; Fatzinger, 2025) indicate that it would require at least 30 years to oxidize all the carbon in peat (used as a substrate) to CO_2_.*

The amount of CO_2_ released from the complete oxidation of the carbon in peat can be calculated. Assuming a dry bulk density of peat at 0.1 g cm^-3^, a cubic meter of peat has a mass of 100 kg. If this was burned for energy, the 50% carbon in the m^3^ of peat would be immediately converted to 183 kg CO_2_ (not CO_2_-equivalent). If the peat decomposed at 1% per year this value would be 1.83 kg CO_2_ per year (or 0.5 kg C per year). This highlights the importance of understanding the rate of microbial decomposition.

**Figure 3:**
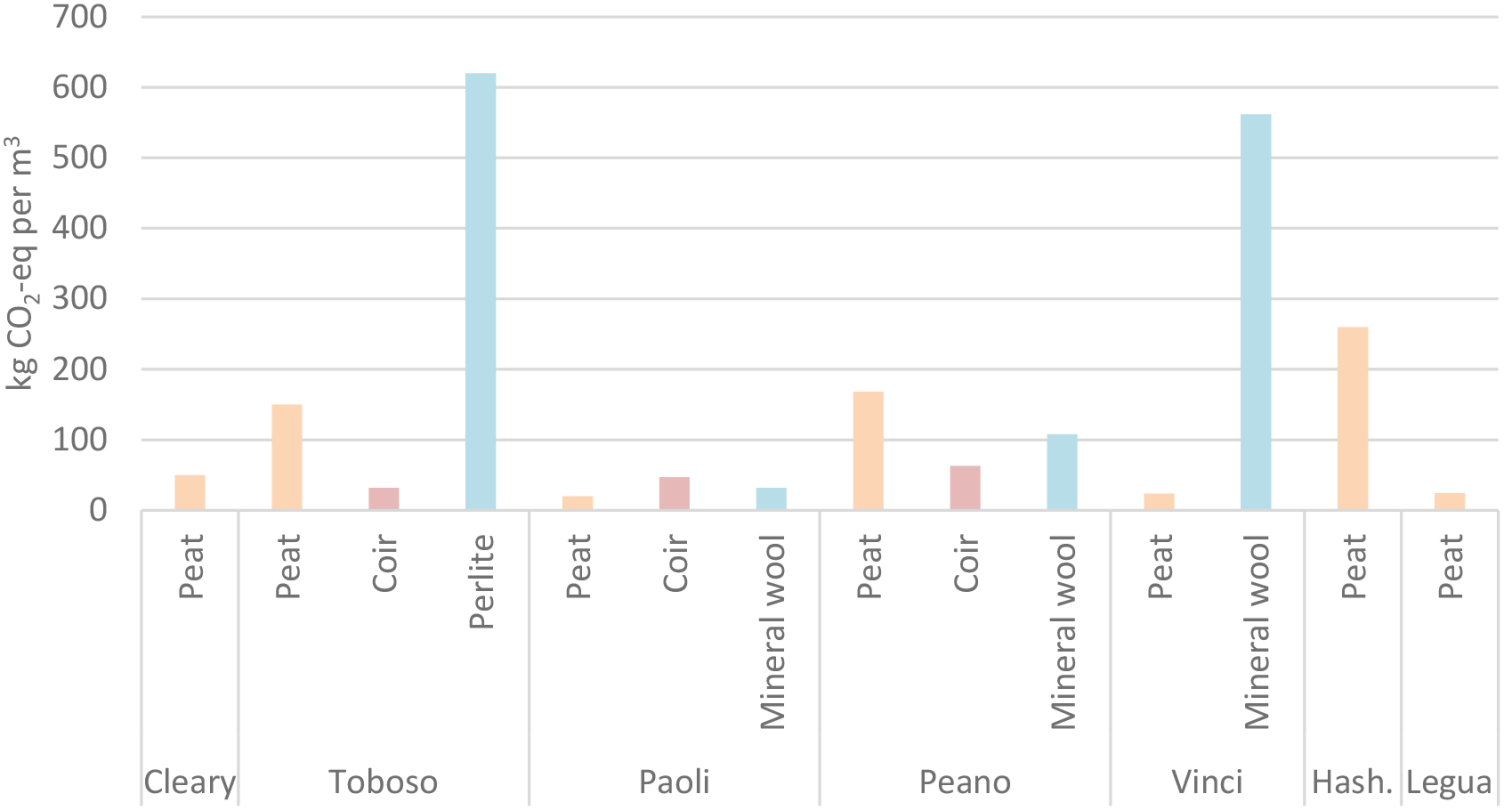
Global warming potential of peat, coir pith, perlite, and rock wool as determined by life cycle assessment across seven studies (Cleary et al., 2005; Toboso-Chavero et al, 2021; Paoli et al., 2022; Peano et al, 2012; Vinci and Rapa, 2019; Hashemi et al., 2024; Legua et al, 2021). Data from all studies were converted to kg CO_2_-eq per m^3^.

Toboso-Chavero et al. (2021) conducted a comparative analysis of peat (harvested in Germany), coir pith (from the Philippines), and perlite (from Türkiye) all transported to Spain (**Figure 3**). They reported that perlite had the greatest GWP with 620 kg CO_2_-eq m^-3^ compared to peat at 150 and coir pith at 32 kg CO_2_-eq m^-3^. In contrast, Paoli et al. (2022) compared peat (from Latvia) to coir pith and rock wool (sources not provided). They reported GWP for peat as 20.2 kg CO_2_-eq m^-3^, compared to coir pith at 47 and rock wool at 32.1 kg CO_2_-eq m^-3^. These authors attribute the high GWP of coir pith to the fertilizer used in coconut production, while Toboso-Chavero et al. (2021) considered coir a by-product and thus did not include coconut production inputs in the LCA calculation. Even without the inclusion of production inputs, the environmental impact of coir pith includes processing to remove salt, tannins, and phenolic compounds. It is often washed with a solution of calcium nitrate to displace sodium and balance the pH. This requires significant amounts of water, which is discharged to waste.

Values of GWP reported in the literature for peat range from 20.2 to 260 kg CO_2_-eq m^-3^ (**Figure 3**) (Cleary et al., 2005; Toboso-Chavero et al, 2021; Paoli et al., 2022; Peano et al, 2012; Vinci and Rapa, 2019; Hashemi et al., 2024; Legua et al, 2021). This 13-fold range highlights the importance of the assumptions and boundaries in the LCA. The GWP of peat is less than 50 kg CO_2_-eq m^-3^ in four of the seven studies.

Peano et al. (2012) reported threefold higher GWP for peat than other studies at 168 kg CO_2_-eq m^-3^. But 50% of this GWP assumed emission of nitrous oxide (N_2_O) after use. The basis for this assumption is unclear. Because nitrous oxide has a GWP 273 times greater than CO_2_ the assumption of incomplete denitrification of nitrate in peat is critical. The transformation of nitrates to N_2_O is favored by oxygen-deprived environments, but peat provides aeration and oxygen diffusivity in container substrates (Schmitz et al, 2013) so denitrification to N_2_O should be reduced. If peat is recycled to the field after use it can increase aeration in soils and potentially reduce N_2_O emissions and N_2_O-related GWP.

Hashemi et al. (2024) reported an unusually high value of 260 kg CO_2_-eq m^-3^ for peat using the same LCA inventory database (Econinvent) as Peano et al. (2012), but attributed 80% of the CO_2_ emissions to rapid peat decomposition after use. They also report the GWP of a wood fiber derived from willow trees with an astonishingly low GWP of 3.4 kg CO_2_-eq m^-3^ with zero emissions from decomposition. These extreme high and low values for decomposition rates make this study an outlier. They do, however, highlight the importance of understanding the rate of decomposition of products following their use in soilless media.

Similar to Peano et al. (2012); Toboso-Chavero et al. (2021) reported an unusually high GWP for peat (150 kg CO_2_-eq m^-3^) but they did not provide enough detail to understand their underlying assumptions.

## Comparison of alternatives based on a functional unit

Media components are not functionally equal. For this reason, LCA’s often define the functional unit (FU) as a representative quantity on which all materials or processes are calculated. Most papers comparing horticultural substrates use a m^3^ (cubic meter) as the FU (Toboso-Chavero et al., 2021; Paoli et al, 2022; Peano et al, 2012; Vinci and Rapa (2019). Legua et al. (2021) used 1 kg of fresh strawberries produced as the FU to compare three substrates including 100% peat, 100% dredged sediment, and equal parts peat and dredged sediment. They found that inputs (energy, fertilizer, pesticides) and outputs (leachate, N_2_O, NH_3_) including GWP were lowest for the peat substrate. Peat required 75% less volume to produce 1 kg strawberries and resulted in 71% less kg CO_2_-eq compared to the dredged sediment substrates. Normalizing the GWP to m^3^ as the FU, the peat substrate in this research resulted in 23.4 kg CO_2_-eq m^-3^ (**Figure 3**), similar to Paoli et al. (2022). Likewise, Ruett et al. (2024) compared peat, coir pith, and stone wool to miscanthus straw substrates using 1 Mg tomato production as the FU. They reported the GWP of peat to be 3x larger than other substrates and attributed this to greater substrate production costs and greater cultivation inputs for peat. These assumptions, however, are contrary to multiple horticultural studies on the yield of crops from peat (Raviv et al., 2019; Ceglie et al., 2015).

## Comparison of alternatives based on overall environmental impact

LCAs also consider environmental, human health, and societal impacts. Toboso-Chavero et al. (2021) measured eight environmental impact categories including: terrestrial acidification, marine eutrophication, ecotoxicity, land use, fossil resource scarcity, and water consumption. They assessed social impacts on community infrastructure, human rights, gender inequality, health and safety, and labor rights. Each of these categories had one or more subcategories. Paoli et al. (2022) provided assessments of four “damage categories” including human health, ecosystem quality, climate change, and resources. They reported these impacts in units of milliEcopoint (mPt), where 1 Pt is representative of one-thousandth of the yearly environmental load of one average European inhabitant. Finally, Vinci and Rapa (2019) evaluated environmental, human health, and economic impact. There were 12 sub-categories of environmental impacts expressed in units of extinct species per year. Eight sub-categories of human health were expressed in disability-adjusted life years, which they described as a measure of overall disease burden expressed as the number of years lost due to ill health, disability or early death. And finally, two sub-categories of economic impact were expressed in USD.

**Figure 4:**
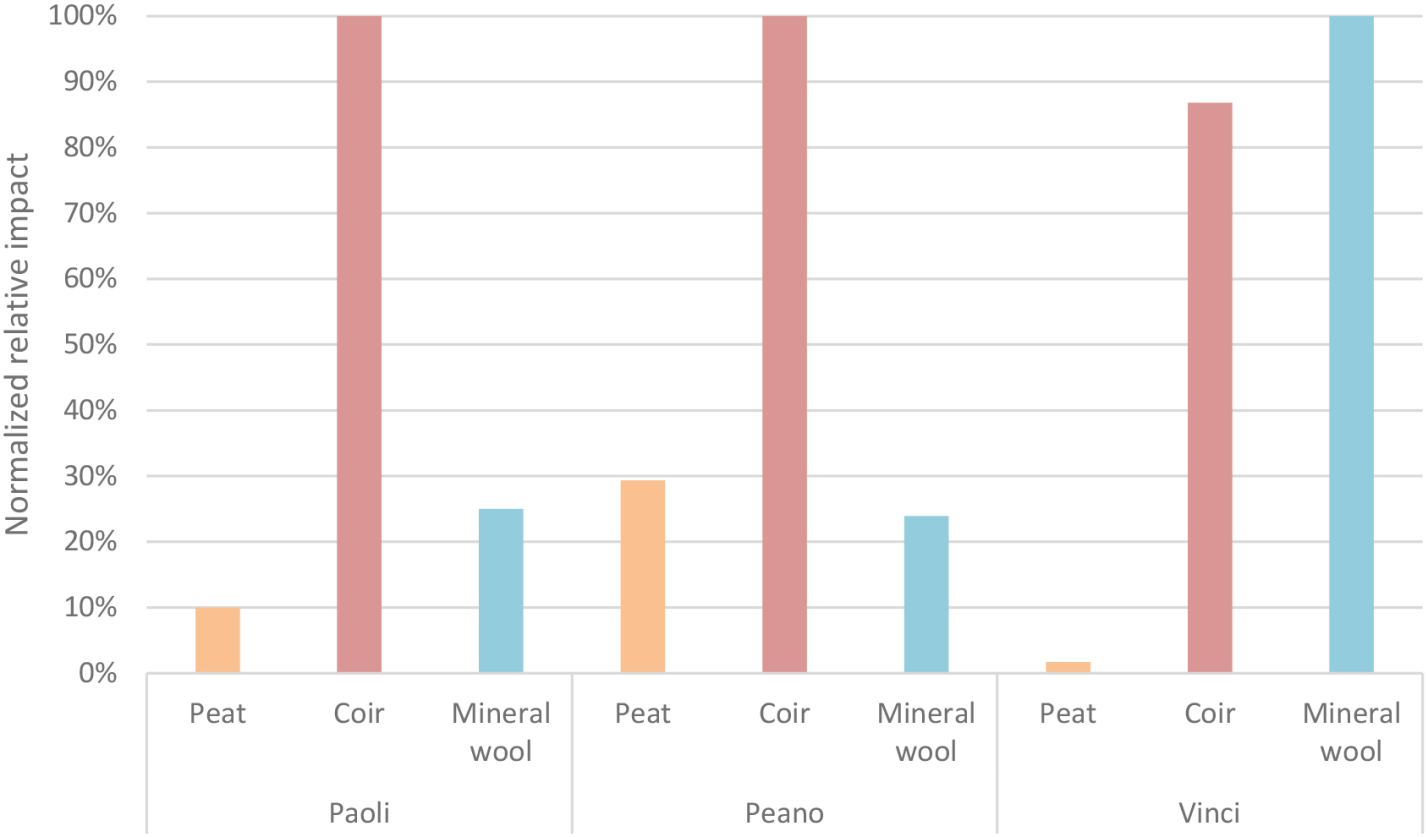
Normalized environmental impact of peat, coir pith, perlite, and rock wool as determined by life cycle assessment across three studies (Paoli et al., 2022; Peano et al, 2012; Vinci and Rapa, 2019).

Variation in assumptions makes comparisons between studies difficult, but relative comparisons among options in the same study can be made. **Figure 4** shows the normalized (percent of highest value) collective environmental impact from three substrate components across three studies (only three of the seven LCA studies provided this broader environmental assessment). In every case, the environmental impact from coir pith was substantially higher than peat. In the Paoli et al. (2022), the impact from coir pith was higher than mineral wool because they included the environmental cost of the retting process to extract coir pith from the husks. This uses large volumes of water. They also included the shipping of coir pith from South Asia to Europe.

The blanket statement that peat is less sustainable than alternatives is not supported by analysis of the LCA literature and careful examination of assumptions regarding CO_2_ emissions.

## Potential to reduce the use of peat in containerized media

Several products can reduce the use of peat but cannot fully replace it (Raviv et al., 2019; Ceglie et al., 2015). The unique physical and chemical properties of sphagnum peat make it the most widely used substrate component in North America and Europe (Nguyen et al., 2026). After processing, peat is flowable, which facilitates combining it with other components. Peat has an ideal combination of air volume (> 20% v/v), high water availability (> 50% v/v) and high water buffering capacity (Michel, 2010). It is stable for greenhouse crop cycles of more than 12 months (Caron and Michel, 2015). Unlike several other alternatives, it has little or no nitrogen immobilization and thus requires less fertilizer inputs (Boyer et al., 2012; Carlile et al., 2015). The chemical properties of peat are also excellent. It has higher cation exchange capacity (CEC) than coir pith and other commonly used substrate components (Carlile et al., 2015; Puustjarvi and Robertson, 1975; Altland et al., 2014), which results in less fertilizer leaching and less pH drift (Argo and Biernbaum, 1997; Rippy and Nelson, 2007).

Peat’s physical and chemical properties have a profound value to root health (Fussy and Papenbrock, 2022). Peat’s porosity and capillarity create a rhizosphere that alternates between water‐filled and air‐filled states; during wet phases, low gas diffusivity and elevated microbial O_2_ consumption depress root-zone O_2_ partial pressure, constraining mitochondrial respiration and adenosine triphosphate (ATP) supply. This suppresses active ion transporters and root growth, thereby reducing nutrient uptake. (Naasz et al., 2005; Schmitz et al., 2013; Ben-Noah & Friedman, 2018). Peat-based media thus require sufficient air-filled porosity (AFP) (Heiskanen, 1997) which is often achieved by mixing peat with coarse components such as perlite. Conversely, when irrigation is limiting and substrates are over aerated, seedling growth declines, indicating hydraulic–aeration tradeoffs: excessive AFP can limit water supply, lowering stomatal conductance and photosynthetic carbon gain. (Boudreault et al., 2023). Mechanistically, peat’s hydraulic conductivity and relative gas diffusivity (D_s_/D_0_) govern O_2_ delivery versus water availability; optimal D_s_/D_0_ in growth substrates lies near 0.01–0.015, aligning aeration with transpiration demand (Schmitz et al., 2013). Chemically, peat exhibits substantial cation exchange capacity (CEC) and variable base saturation, which buffer pH and modulate nutrient speciation (e.g., Fe, Mn, Zn), directly influencing uptake kinetics and photosynthetic enzyme cofactor status (Rippy and Nelson, 2007). Organic functional groups in peat also complex multivalent cations; molecular-scale interactions and aging reorganize the peat matrix, altering ion binding and mobility, with implications for micronutrient bioavailability at the root interface (Kunhi Mouvenchery et al., 2012). Integrating these properties, peat affects crop physiology via (i) O_2_ supply constraints on root respiration under high saturation, (ii) hydraulic limitations on stomatal function and photosynthesis under over aeration, and (iii) CEC/complexation driven control of nutrient activity at the root surface. Optimizing irrigation to maintain favorable AFP and D_s_/D_0_ while managing pH and ion balances is therefore central to sustaining nutrient uptake and photosynthetic performance in peat-based media (Naasz et al., 2005; Boudreault et al., 2023; Rippy and Nelson, 2007). Ceglie et al. (2015) used response surface analysis to estimate the optimum ratios of green compost, palm fiber, and peat for production of tomato. Their data indicate that tomatoes require at least 20% peat and lettuce required 60% peat for optimum growth.

Sawdust from sawmill or furniture manufacturers is being used but requires additional fertilizer to prevent nutrient immobilization caused by the microbial decomposition of the wood. Disc-refined or extruded wood fibers are increasingly used in Europe and North America (Nguyen et al., 2026). Peat can be replaced up to 25% v/v using wood fibers with acceptable plant performance, though nitrogen immobilization and physical stability limit greater replacement rates (Sradnick et al., 2023; Sradnick and Körner, 2025). Across greenhouse trials, wood fiber blends typically increase air space and lower water-holding capacity, and often require higher N rates to offset immobilization and maintain crop performance (Thiessen et al., 2024). For extruded fibers, twin screw extrusion yields peat like, pathogen free fibers but with lower water holding capacity than peat (Dittrich et al., 2021). Like peat, the use of wood products requires sustainable management of natural resources (forests).

Containerized media can not only be recycled but reused. One of the frontiers of controlled environment agriculture is multiple planting in the same media. The biological stability of peat facilitates this reuse, sometimes over multiple crop cycles (Fatzinger, 2025).

Soilless media is a type of hydroponic culture as plants are irrigated (fertigated) with a dilute nutrient solution each time they are watered. But some hydroponic production systems directly submerge roots in a solution that eliminates the need for soilless media. The nutrient film technique (NFT) and deep-water culture (DWC) are common types of this production, but neither allows marketing the entire plant. Liquid hydroponic systems have been historically wasteful of water and nutrients because the solution is often discarded at frequent intervals, but a mass balance approach can be used to minimize the discharge of solution to the environment (Langenfeld et al., 2022).

## Potential of peat used for horticulture to reduce global warming potential

Reforestation has the potential to provide a 10 Gt CO_2_-eq per yr CO_2_-eq reduction globally, while agricultural, grassland, and wetland conservation strategies can provide less than 2 Gt CO_2_-eq per yr (Griscom et al., 2017). In the US, Cook-Patton et al. (2020) estimated there are 51.6 Mha of land that could be reforested capture 0.34 Gt CO_2_-eq per yr without adversely affecting cropland and urban areas. To reforest the US, the forest seedling industry would have to double its current output. In-ground, bareroot nurseries (Fargione et al., 2021) have been used for seedling propagation but growth is reduced. The fastest way to ramp up US capacity is through container production, and peat is generally the media of choice.

## Increasing global demand for soilless media

Global demand for soilless media is predicted to grow from 116 Mm^3^ used in 2022 by 250% to 400% in 2050 to reach a total demand of 290 to 464 Mm^3^ (Blok et al., 2021; Nguyen et al., 2026). The area of covered cultivation in China alone has grown from 700,000 ha to over 3,000,000 ha in the past five years. This global increase in high-input agriculture will increase the demand for all soilless media. Canada would have to increase production of peat 3.4-fold just to replace current European extraction rates and this could quadruple over the next 25 years. Global market prices will determine global demand. Canada will need to retain its focus on sustainability to supply peat for an international market.

Russia also has large peat reserves with 1.39 × 10^6^ km^2^ peatlands (Kopansky et al., 2022), 33% of the global total. During the Soviet era, Russia extracted peat for energy and used it as a fertility source in agriculture (Panov and Misnikov, 2015), with peak extraction rates reaching 220 Mt in 1975. However, the transition to natural gas and mineral fertilizers, coupled with the cessation of state subsidies, led to a dramatic decline to 2 Mt by 2011. Global politics and current sanctions will impact peat availability from Russia in the immediate future, but the scale of its peatlands will make it an important resource for satisfying the increasing global demand.

In Chile, the Peatland Protection Law enacted in 2024 aimed to safeguard peatlands by prohibiting extraction and degradation. While the law was intended to ensure the conservation of Chilean peatlands, early drafts of the law prohibited the use of any peat. Later revisions allowed for sustainably sourced peat for horticulture.

Coir pith has been identified as a high-quality alternative to peat (Blok et al., 2021), but as a byproduct of the coconut industry, its availability is limited by coconut production. Gbollie et al. (2021) estimates that of the 62.8 Mt global coconut production, only 10% of husks are being used for horticultural coir pith resulting in 1.5 Mt. Assuming a bulk density of 125 kg m^-3^, there is currently 12 Mm^3^ coir pith on the market today. Nguyen et al. (2026) similarly estimated a current supply of 11.3 Mm^3^ coir pith available globally and as much as 64 Mm^3^ available annually by the year 2050. A potential of 120 Mm^3^ coir pith would be available if the entire global harvest could be utilized for horticulture (an unlikely scenario). Even this 120 Mm^3^ per year would not be sufficient to supply the projected 290 to 464 Mm^3^ of substrate needed per year by 2050 (Blok et al., 2021; Nguyen et al., 2026).

## Conclusion

The history of agriculture is filled with advances that have increased food production. Optimizing plant growth in soilless media has been a central part of these yield increases. The physical and chemical properties of peat make it uniquely valuable to the optimization of the root zone. More people are living in urban areas and green, livable cities and substrates are essential for responsible landscape beautification. Approximately 68% of peat-based substrates in North America are used by professional growers and 32% are used by home gardeners (Nguyen et al., 2026). Both uses provide economic, environmental, and human health benefits (Gill et al, 2007; Hall and Dickson, 2011; Langellotto and Gupta, 2012).

The disparity between North America and Europe means that sustainable solutions will require different approaches. North American peatlands are vast and in excellent health. The small fraction used for horticultural peat has minimal environmental impact because extracted areas can be returned to functioning peatlands in a short period of time. This potential for long-term sustainability of peatlands in combination with the smaller GWP and environmental impacts (from LCA) make horticultural peat a valuable resource for the future of controlled environment agriculture.

This review will help policymakers and stakeholders recognize that peatland use in North America, and potentially other locations, is sustainable and a critical resource for our horticultural industries and food supply.

## Footnotes

1 Disturbed Canadian peatlands are caused by agricultural crops (63%), mining (18%), hydroelectric reservoirs (12%), forestry (3%), roads, rail lines, etc. (3%), powerlines (1%), and horticultural peat extraction (1%)^19^.

2 *1 gigaton is equal to 1 petagram, or 10^15^ g*.

3 A CO_2_-eq is a unit of measurement that sums the warming effect of all greenhouse gases into a single value equivalent to amount of CO_2_ needed for the same warming effect.

4 This is approximately double the land area of Texas.

5
*In persistent decline or cannot fully recover within decadal timescales.*

6 Dual layers of a functioning peatland with an acrotelm or upper layer of high hydraulic conductivity and fluctuating water table over a catotelm or lower layer that is more inert and permanently saturated.

7 Assumes 4.4 tons dry peat equals 1 ton oil equivalent, and the bulk density of dry peat is 350 kg m^-3^.

8 Mineral wool is a general word for describing glass wool (produced from sand) and rockwool or stonewool (produced from basaltic rock).
